# Pulmonary embolism with junctional tachycardia: A serious complication after COVID-19 vaccination

**DOI:** 10.1016/j.amsu.2022.103983

**Published:** 2022-06-28

**Authors:** Chaymae miri, Amine Bouchlarhem, Soumia boulouiz, Noha El ouafi, Zakaria Bazid

**Affiliations:** a Faculty of Medicine and Pharmacy, Mohammed I^st^ University, Oujda, Morocco; b Department of Cardiology, Mohammed VI University, Hospital Mohammed I University, Oujda, Morocco

**Keywords:** COVID-19, Vaccine, Pulmonary embolism, Pharmacovigilance

## Abstract

**Introduction:**

the association between the development of a thromboembolic event following COVID-19 vaccination is very rare, it represents less than 0.1% of vaccinated cases. Until now this association remains to be discussed.

**Case presentation:**

A 49-year-old man presented to the Emergency Department a 7-day after receiving her second dose of BNT162b2 mRNA COVID-19 (Pfizer-BioNTech), and he was diagnosed with pulmonary embolism (PE) with junctional tachycardia on ECG. The biological workup showed an increase in CRP with elevated D-dimer, but no abnormalities in cardiac markers, including troponin and BNP, the COVID-19 testing was negative and absence of thrombocytopenia. The patient was put under curative anticoagulation by rivaroxabon.

**Discussion:**

Studies have reported the association of venous thrombosis after administration of the COVID-19 vaccine with negative FP4 antibodies and normal platelet count which is similar with our patient. Moreover, spike proteins generated by mRNA vaccines can produce a pro-inflammatory state, a cascade of events guiding to endothelial dysfunction and afterwards to the development of venous thrombosis.

**Conclusion:**

All the same that some studies association COVID-19 immunizations to the development of VTE, we nevertheless recommend COVID-19 vaccination, due to the rarity of these events, compared to the hypercoagulable effects and other serious complications of COVID-19 infection.

## Highlights

• The association between the development of a thromboembolic event following COVID-19 vaccination is very rare.
• Studies have reported the association of venous thrombosis after administration of the COVID-19 vaccine with negative FP4 antibodies and normal platelet count which is similar with our patient.
• We nevertheless recommend COVID-19 vaccination, due to the rarity of these events, compared to the hypercoagulable effects and other serious complications of COVID-19 infection.

## 1 Introduction

Severe Acute Respiratory Syndrome Coronavirus 2 (SARS-CoV-2) was declared a global health emergency and it was having a negative impact on the global economy. COVID-19 vaccines were developed as a result of coordinated global efforts, they were intended for public administration and are now widely available [1]. The vaccines have been shown to be safe and effective in reducing serious infections, hospitalizations and deaths [2,3]. However, some cardiovascular adverse effects have been demonstrated in the situation of SARS-CoV-2 RNA vaccination, such as cerebral venous sinus thromboembolism, portal vein thrombosis and lower limbs and among them pulmonary embolism. (EP) [4].

With the frequency number of suspected COVID vaccine-associated thrombosis rises, we report extensive thromboembolism in a 59-year-old man that occurred 7 days after receiving the first dose of the BNT162b2 Pfizer-BioNTech mRNA vaccine.

## 2 Case presentation

**Fig. 1:**
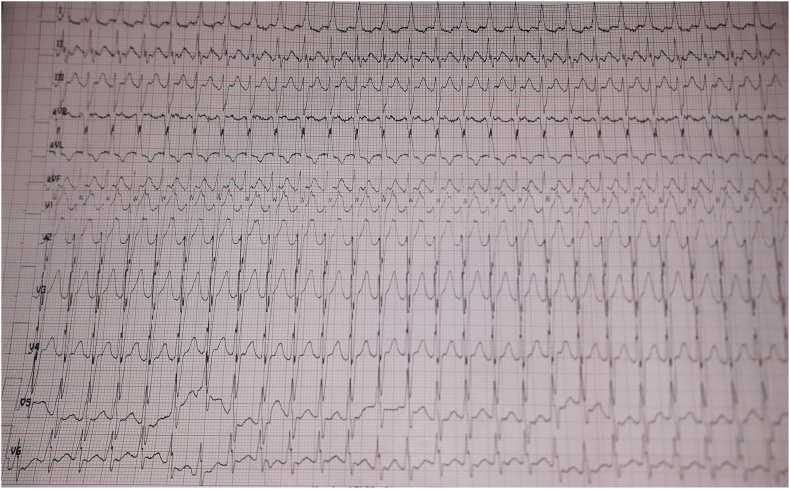
ECG shows a regular fine QRS tachycardia with retrograde P waves in favor of a junctional tachycardia.

**Fig. 2:**
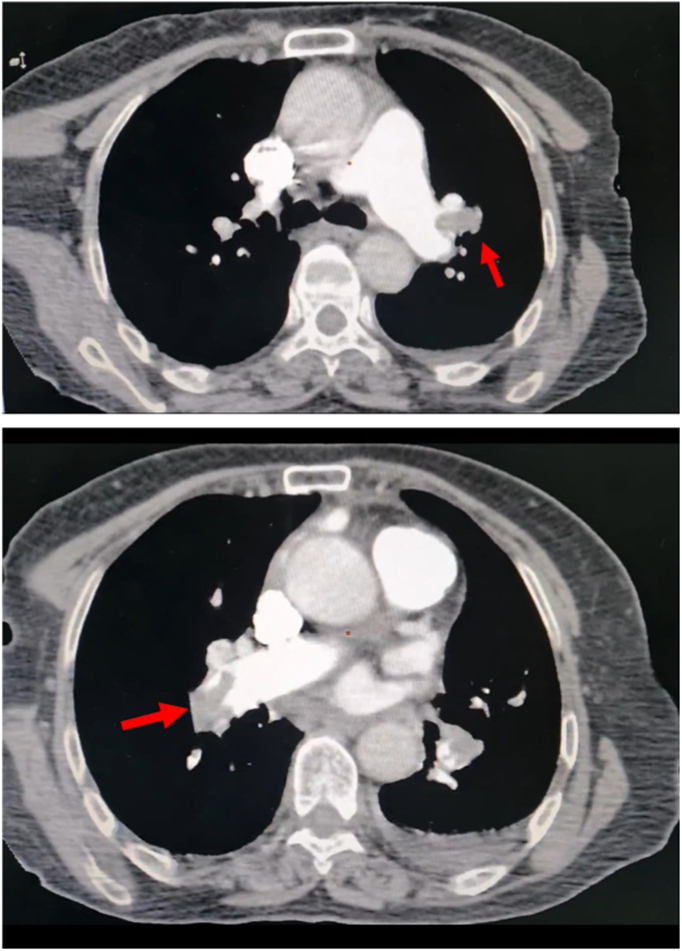
Chest CT scan with contrast injection showing hypodense material in both pulmonary arteries (red arrow) in favor of a pulmonary embolism. (For interpretation of the references to colour in this figure legend, the reader is referred to the Web version of this article.)

We report the case of a 49-year-old man, with no medical history, admitted to the emergency department for the management of acute respiratory failure 7 days after receiving the second dose of the Pfizer COVID-19 vaccine 4 days before. On admission, the patient was hemodynamically and neurologically stable, dyspneic with a respiratory rate of 28 cpm/min and a room air SpO2% of 92%, T° = 38.7, weight 95kg with a height of 1.76cm and a BMI of 31.96kg/m2. The examination was unremarkable. ECG found a junctional tachycardia with a HR at 156 bpm (Fig. 1), An echocardiography is performed objectifying an aspect of acute pulmonary heart with dilatation of the right cavities with right ventricular dysfunction. Given the suspicion of a pulmonary embolism with an intermediate clinical probability (Welles score of 4.5) with an increase in D-dimer levels, a thoracic angioscanner with injection was performed confirming the presence of a proximal right pulmonary embolism (Fig. 2). The biological workup showed an increase in CRP, but no abnormalities in cardiac markers, including troponin and BNP, the COVID-19 testing was negative and absence of thrombocytopenia.

The history did not indicate any recent bed rest, prolonged travel or surgery in the last 3 months, and given the notion of vaccination and the reports published in this sense, a causal link was made between the vaccination and the pulmonary embolism.

The patient was put under curative anticoagulation by rivaroxabon, with a good initial clinical evolution, the junctional tachycardia was regressed spontaneously in sinus rhythm. A report was made to the pharmacovigilance center. and the patient is discharged home and he is followed in consultation with a control 3 months after his release.

## 3 Discussion

In the fight against the COVID-19 pandemic, a collective vaccination campaign is critical. Several vaccines, including Pfizer's (BNT162b2mRNA), Moderna's (mRNA-1273), J&J's (Ad26.COV2.S), and AstraZeneca's (AZD1222), have been approved as a result of these efforts. Nevertheless, adverse effects have been observed following vaccine injections. Rare undesirable damage has been reported such as myocarditis, pericarditis, vascular syndromes, thrombosis complication and other diseases [[5], [6], [7], [8], [9]].

The association between COVID-19 vaccine and thromboembolic complication is increasingly demonstrated in the literature. The timing of vaccination with the development of thrombosis is nonspecific. Thrombosis can develop within the first 7–10 days [10]. Our case involves a patient who had PE 7 days after receiving the second dose of the Pfizer vaccination and who had no other recognized risk factors for developing PE. Several nations have documented a rare thromboembolic events, more often deep venous thrombosis (DVT) and pulmonary embolism (PE), which occurred 7–14 days following vaccination with specific COVID-19 vaccines and were either linked or not with thrombocytopenia [11].

Scientific research are discussing the relevance of vaccine-induced thrombotic thrombocytopenia (VITT) in the thrombosis episodes after COVID-19 vaccination. The VITT condition intently similar to heparin-induced thrombocytopenia (HIT) [12], because it characterizes by a positive anti-platelet factor 4 (PF4) IgG antibodies. However, some cases show that VITT can occur following receiving the mRNA-1273 vaccination [13,14]. Unfortunately, the factor 4 was not verified in our patient to assess the VITT.

Some studies have discussed the resemblance between COVID-19 vaccines and virus COVID-19. COVID-19 infection leads to a major thrombotic state proven by several studies, the same thrombogenic effect can occur after vaccination, the mechanism of which remains unknown until now. Moreover, spike proteins generated by mRNA vaccines can produce a pro-inflammatory state, a cascade of events guiding to endothelial dysfunction and afterwards to the development of venous thrombosis [15].

Indeed, our patient had no risk factor predisposing to the development of acute venous thrombosis, in particular pulmonary embolism, and he tested negative for COVID-19 infection, however the development of this thrombosis due to the mRNA-1273 vaccine is the most reasonable explanation. The formation of emboli may be secondary to a cause other than VITT, with a predilection for the venous circuit after mRNA vaccination. Studies have reported the association of venous thrombosis after administration of the COVID-19 vaccine with negative FP4 antibodies and normal platelet count which is similar with our patient [15,16].

The SCARE guidelines were used in the writing of this paper [17].

## 4 Conclusion

Despite the fact that some studies association COVID-19 immunizations to the development of VTE, we nevertheless recommend COVID-19 vaccination, due to the rarity of these events, fewer than 0.01% of the overall vaccinated population has been affected. Compared to the hypercoagulable effects and other serious complications of COVID-19 infection are well established by several studies and in the literature. further research is needed to better assess the causality and specificity between vaccine-induced thromboembolism and take into account patient characteristics.

## Consent for publication

Written informed Consent was obtained from the Child's patients for publication of this case report and accompanying images. A copy of the written consent is available for review by the Editor-in-Chief of this journal on request.

## Funding

This research did not receive any specific grant from funding agencies in the public, commercial, or not-for-profit sectors.

## Ethical approval

The ethical committee approval was not required give the article type (case report).However, the written consent to publish the clinical data of the patients was given and is available to check by the handling editor if needed.

## Sources of funding

None.

## Author contributions

CHAYMAE MIRI: Study concept or design, data collection, data analysis or interpretation, writing the paper.

AMINE BOUCHLARHEM: Data collection, data analysis.

SOUMIA BOULOUIZ: Data collection, data analysis.

NOHA EL OUAFI: supervision and data validation.

ZAKARIA BAZID: supervision and data validation.

## Trial registry number

This is not an original research project involving human participants in an interventional or an observational study but a case report. This registration is was not required.

## Guarantor

Chaymae Miri.

## Consent

Written informed Consent was obtained from the patients for publication of this case report and accompanying images. A copy of the written consent is available for review by the Editor-in-Chief of this journal on request.

## Provenance and peer review

Not commissioned, externally peer reviewed.

## Declaration of competing interest

None.
